# Author Correction: FRQ-CK1 interaction determines the period of circadian rhythms in *Neurospora*

**DOI:** 10.1038/s41467-019-13862-3

**Published:** 2020-01-14

**Authors:** Xiao Liu, Ahai Chen, Angélica Caicedo-Casso, Guofei Cui, Mingjian Du, Qun He, Sookkyung Lim, Hang J. Kim, Christian I. Hong, Yi Liu

**Affiliations:** 1 Department of Physiology, University of Texas Southwestern Medical Center, Dallas, TX 75390-9040 USA; 2 State Key Laboratory of Mycology, Institute of Microbiology, Chinese Academy of Sciences, Beijing, 100101 China; 3 Key Laboratory of Biopesticides and Chemical Biology, Ministry of Education, Fujian Agriculture and Forestry University, Fuzhou, Fujian 350002 China; 4 Department of Mathematics, Universidad del Valle, Cali, 760032 Colombia; 5 State Key Laboratory of Agrobiotechnology and MOA Key Laboratory of Soil Microbiology, College of Biological Sciences, China Agricultural University, Beijing, 100193 China; 6 Department of Mathematical Sciences, University of Cincinnati, Cincinnati, 45221 USA; 7 Division of Statistics and Data Science, University of Cincinnati, Cincinnati, 45221 USA; 8 Department of Pharmacology and Systems Physiology, University of Cincinnati, Cincinnati, OH 45267 USA; 9 Division of Developmental Biology, Cincinnati Children’s Hospital Medical Center, 3333Burnet Avenue, Cincinnati, USA

**Keywords:** Circadian rhythms, Fungal physiology, Phosphorylation

Correction to: *Nature Communications* 10.1038/s41467-019-12239-w, published online 25 September 2019.

The original version of this Article omitted the following from the last sentence of the Acknowledgements:

A.C.-C. was supported by Universidad del Valle via the research project CI 71033.

This has now been corrected in both the PDF and HTML versions of the Article

