# Supplementary material for: FRQ-CK1 interaction determines the period of circadian rhythms in Neurospora
Source: Nat Commun. 2019 Sep 25;10:4352. doi: 10.1038/s41467-019-12239-w (PMC6761100; doi:10.1038/s41467-019-12239-w)
Supplement: Supplementary file 1 — Supplementary Information [file 41467_2019_12239_MOESM1_ESM.pdf]

Supplementary Information

FRQ-CK1 interaction determines the period of circadian rhythms in *Neurospora*

Liu et al.

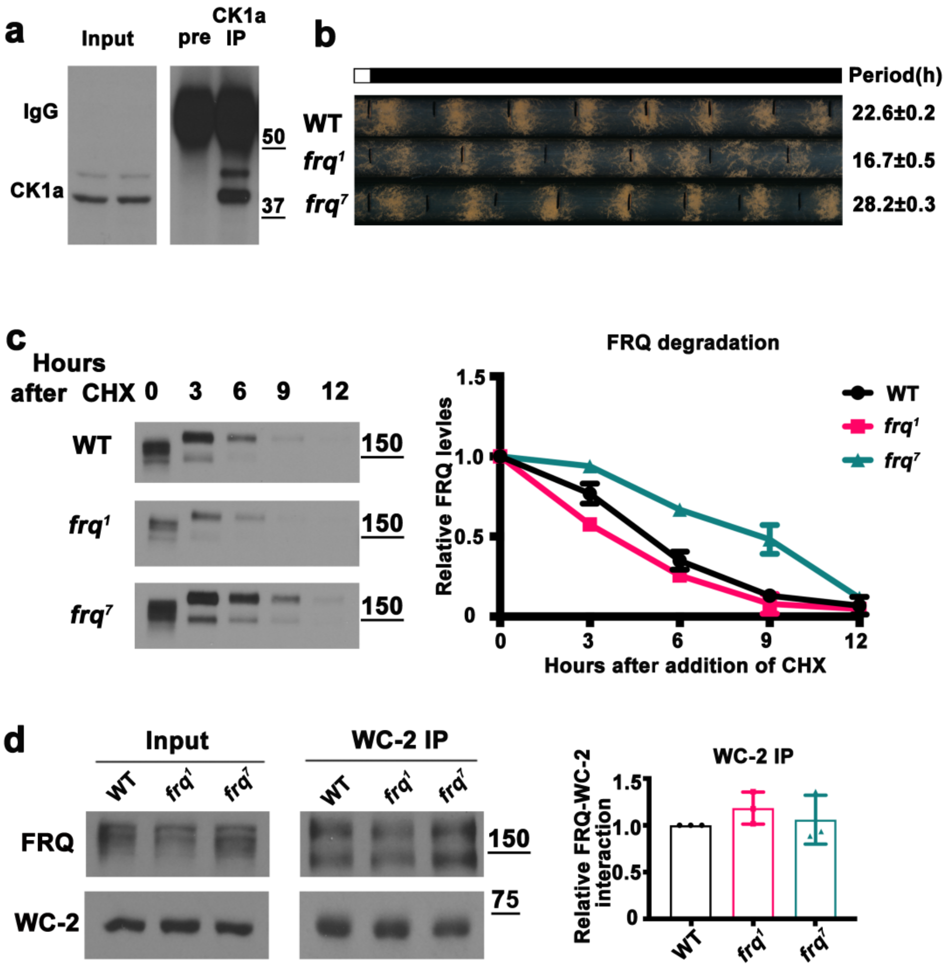

Supplementary Figure 1. Molecular characterization of the *frq*^{1} and *frq*^{7} strains. (a) Analysis of proteins precipitated from *Neurospora* extract with CK1a antibody demonstrates recognition of endogenous CK1a protein. (b) Race tube assay showing period changes and of *frq*^{1} and *frq*^{7} strains. Error bars are standard errors of means (n=4).(c) Western blot analysis for FRQ in the *frq*^{1} and *frq*^{7} strains at indicated times after CHX addition. The densitometric analysis is shown on the right (n=3). (d) Immunoprecipitation of WC-2 in wild-type, *frq*^{1}, and *frq*^{7} extracts. The densitometric analysis in the wild-type strain is shown on the right. Error bars are standard deviations (n=3). Source data are provided as a Source Data file.

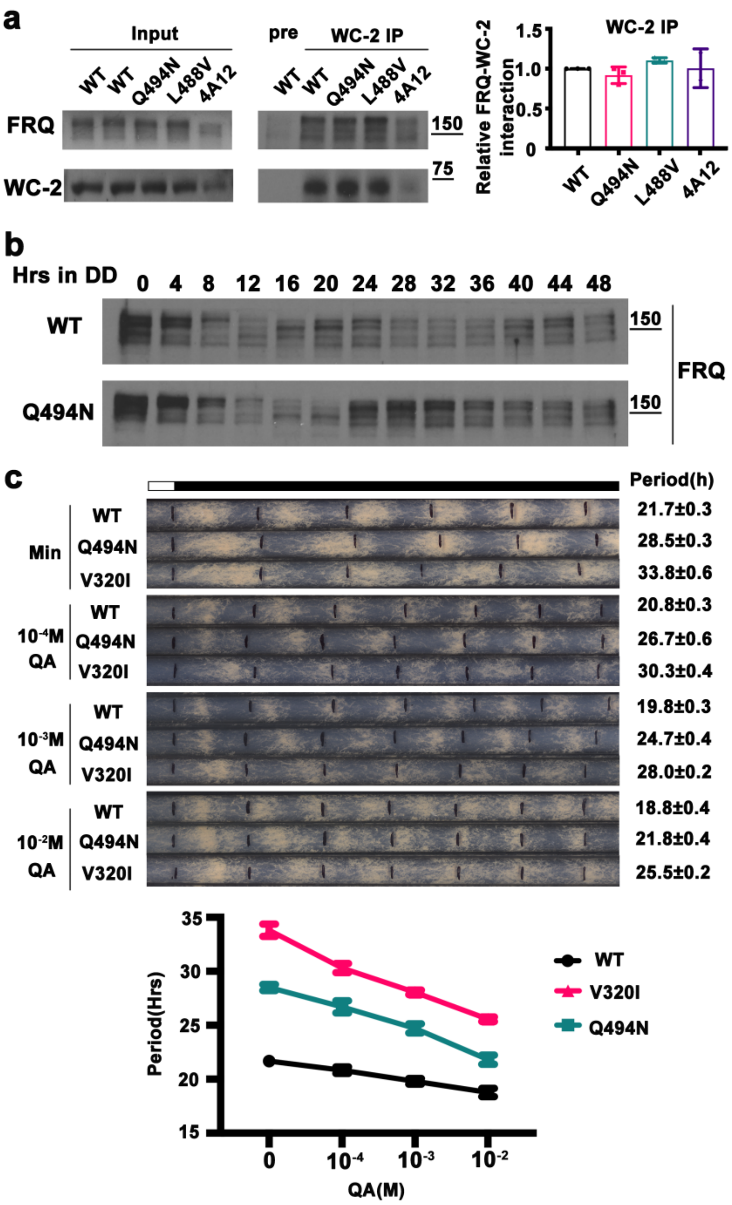

Supplementary Figure 2. FRQ-CK1a interaction dynamics are important for circadian period length. (a) WC-2 immunoprecipitation assays of the indicated strains. The densitometric analysis quantifying the amount of FRQ relative to the amount in the wild-type strain is shown on the right. Error bars indicate standard deviations (n=3). (b) Western blot analysis showing the circadian rhythms of FRQ expression in constant darkness in the indicated strains. (c) Race tube assays showing circadian conidiation rhythms in the indicated strains after induction of CK1a by QA at different concentrations. Quantification of period length of the strains are shown below. Errors are standard errors of means (n=4). Source data are provided as a Source Data file.

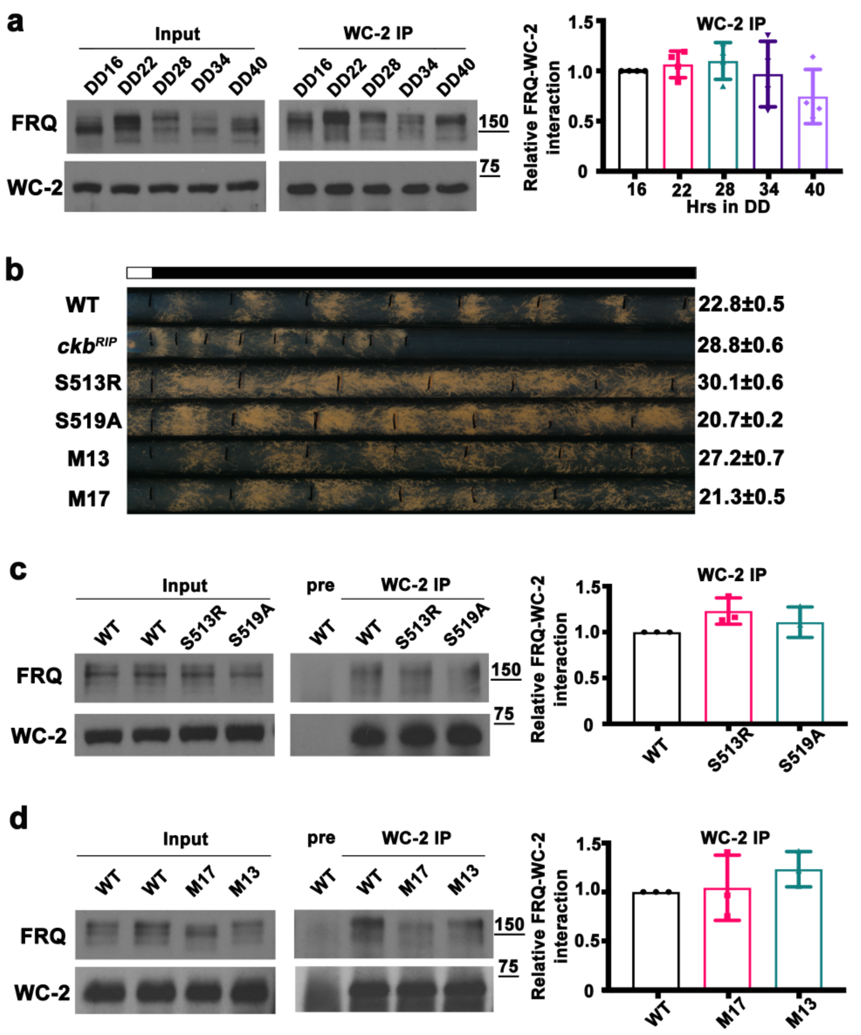

Supplementary Figure 3. FRQ-WC interaction was not affected in period mutants. (a) WC-2 immunoprecipitation assays of the indicated strains grown in LL. The densitometric analysis quantifying the amount of FRQ relative to the amount in the wild-type strain is shown on the right. Error bars indicate standard deviations (n=3). (b) Race tube assay showing circadian conidiaiton rhythms of *ckb*^{RIP}, S513R, S519A, M13, and M17 strains. Errors are standard errors of means (n=5). (c-d) WC-2 immunoprecipitation assays of S513R, S519A, M13, and M17 strains. The densitometric analysis quantifying the amount of FRQ relative to the amount in the wild-type strain is shown on the right. Error bars indicate standard deviations (n=3). Source data are provided as a Source Data file.

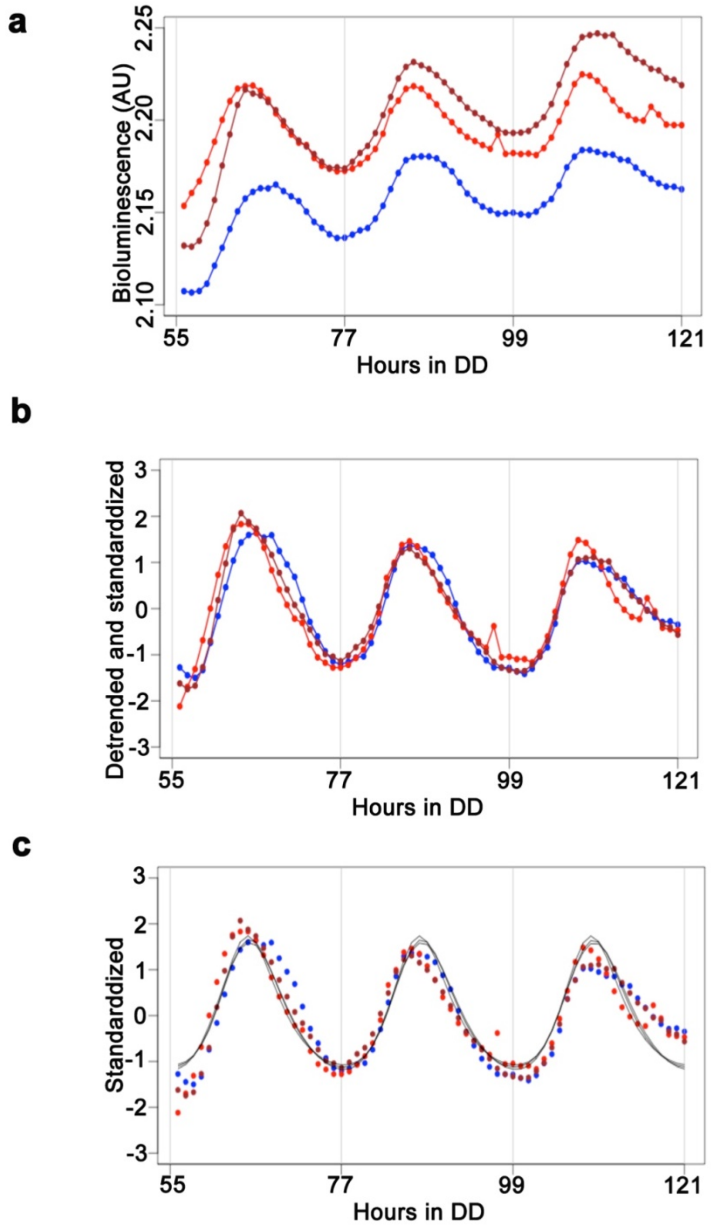

Supplementary Figure 4. Fitting of the experimental data prior to parameter estimation and parameter estimation based on three independent experiments. (a) Bioluminescence measurements from a luciferase reporter fused to the *frq* promoter (n=3). (b) The estimated trend is removed from the original expression level, then standardized. (c) Oscillation profiles from 3 parameter sets (solid curves) overlaid on the standardized expression level of *frq* gene expression measured by *frq*-luciferase reporter (dots). Three curves corresponding to the 3 parameter sets drawn from the MCMC chain have identical shapes.

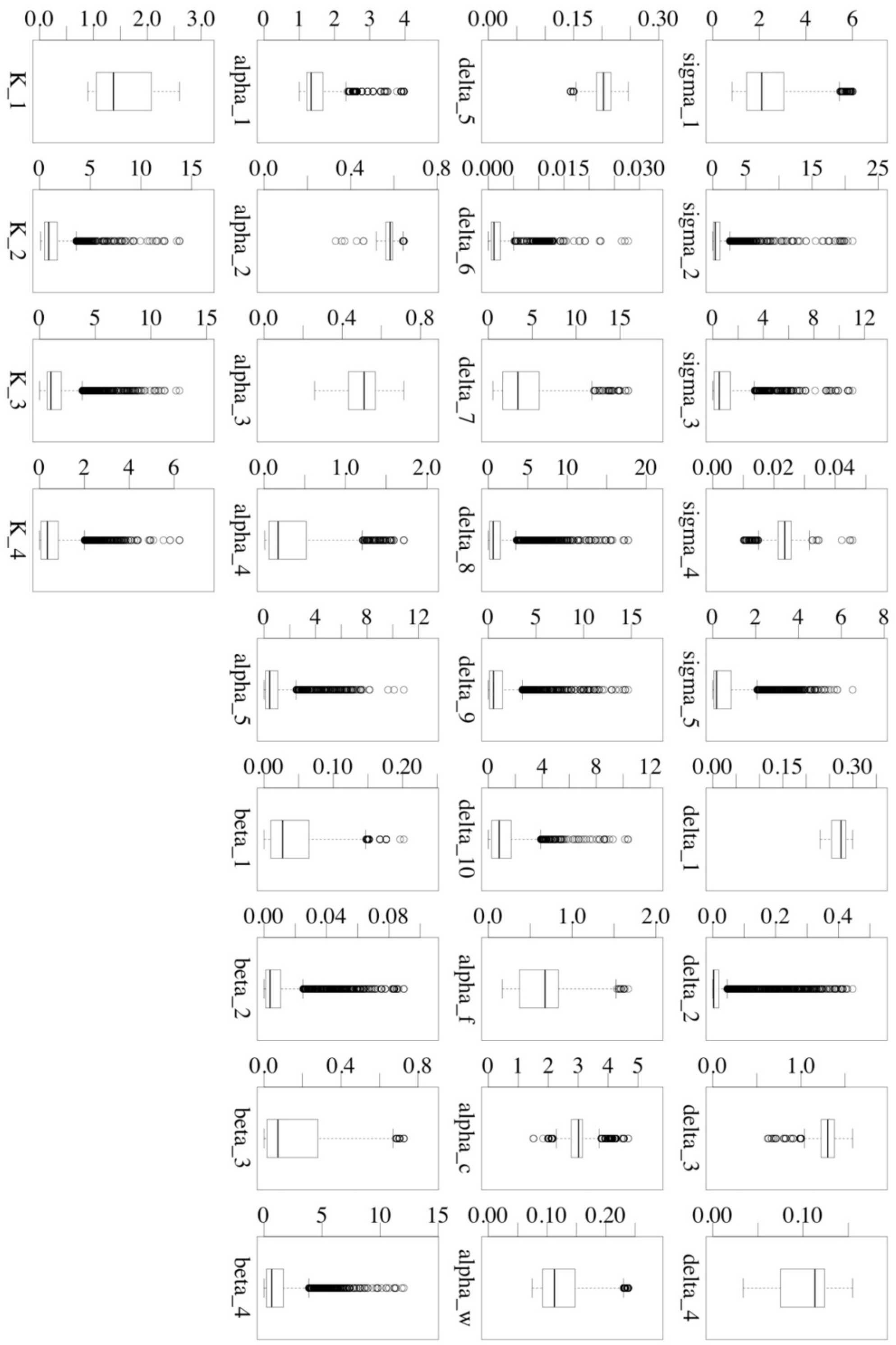

Supplementary Figure 5. Marginal distribution of 31 model parameters drawn from a MCMC chain with 4,000 iterations. Y-axis show numerical values of each parameter in their respective units as described in Table S1.

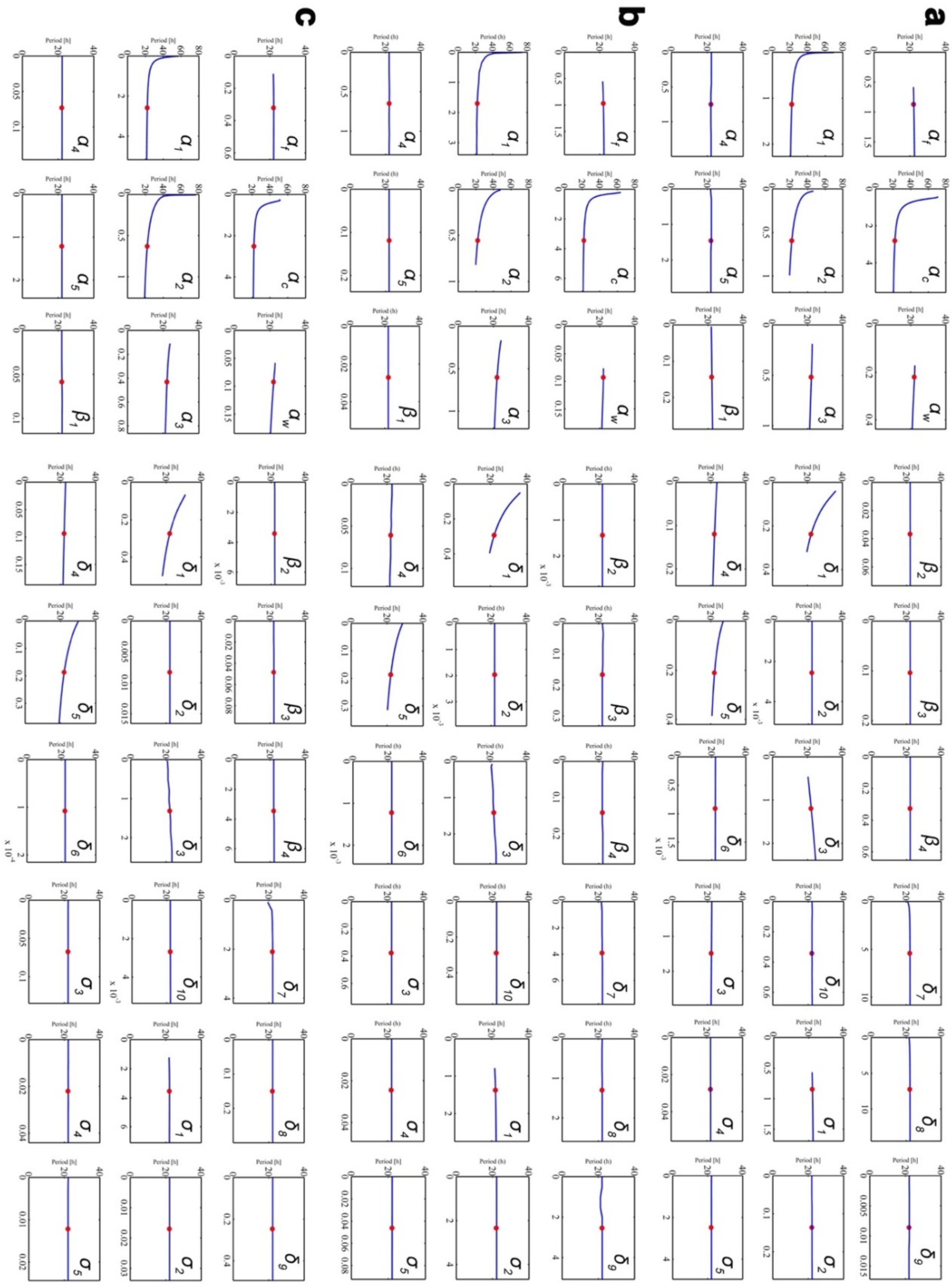

Supplementary Figure 6. One-parameter bifurcation analyses for three sets of parameters. (a-c) One-parameter bifurcation analysis for parameters from (a) set 1, (b) set 2, and (c) set 3. Parameter values are given in Table S1.

Supplementary Table 1. Values, units, and descriptions of parameters used in mathematical modeling.

Parameters	Set 1	Set 2	Set 3	Units	Description
α_f	0.870878612	0.969599683	0.320611907	h^{-1}	Rate of synthesis of FRQ
α_c	2.804900175	3.447119953	2.511403244	$a.u.h^{-1}$	Rate of synthesis of CK1
α_w	0.219706956	0.093004735	0.092404435	$a.u.h^{-1}$	Rate of synthesis of WCC_a
α_1	1.132575798	1.701458277	2.585217081	$(a.u.)^{-1}h^{-1}$	Rate of association between FRQ and CK1
α_2	0.593871864	0.555047615	0.629418704	h^{-1}	Rate of phosphorylation of FRQ:CK1 complex
α_3	0.516499619	0.603515817	0.431117198	$(a.u.)^{-1}h^{-1}$	Rate of association between FRQ:CK1 and WCC_a
α_4	0.748625166	0.649159884	0.073014737	h^{-1}	Rate of phosphorylation of FRQ:CK1:WCC_a
α_5	1.458350375	0.118755742	1.21208905	h^{-1}	Rate of dissociation between FRQ, CK1, and WCC_i
β_1	0.143704179	0.027155705	0.058611284	h^{-1}	Rate of dissociation between FRQ and CK1
β_2	0.036725581	0.001423979	0.003421335	h^{-1}	Rate of dephosphorylation of FRQ:CK1 complex
β_3	0.103946717	0.166316173	0.048470728	h^{-1}	Rate of dissociation between FRQ:CK1 and WCC_a
β_4	0.326428518	0.141742623	3.457019544	h^{-1}	Rate of dephosphorylation of FRQ:CK1:WCC_a
δ_1	0.237607683	0.29278174	0.273954198	h^{-1}	Rate of degradation of *frq* mRNA
δ_2	0.002567856	0.00196427	0.008292166	h^{-1}	Rate of degradation of FRQ
δ_3	1.195795179	1.412607585	1.312519195	h^{-1}	Rate of degradation of CK1
δ_4	0.119505827	0.060763961	0.093592661	h^{-1}	Rate of degradation of FRQ:CK1 complex
δ_5	0.210581592	0.186619481	0.186823146	h^{-1}	Rate of degradation of phosphorylated FRQ:CK1 complex
δ_6	0.000908462	0.001222758	0.000107667	h^{-1}	Rate of degradation of active WCC
δ_7	5.4230748	3.912102529	2.103746067	h^{-1}	Rate of degradation of FRQ:CK1:WCC_a complex
δ_8	7.25089941	1.298127664	0.149940849	h^{-1}	Rate of degradation of FRQ:CK1:WCC_i complex
δ_9	0.008603263	2.542465713	0.247220332	h^{-1}	Rate of degradation of phosphorylated FRQ
δ_{10}	0.342629474	0.289466702	0.002680827	h^{-1}	Rate of degradation of

					inactive WCC
σ_1	0.846786705	1.373112679	3.548666189	$a.u.h^{-1}$	Transcription rate of *frq* mRNA by WCC_a
σ_2	0.136243263	2.340420329	0.017017841	$a.u.h^{-1}$	Transcription rate of *frq* mRNA by FRQ:CK1:WCC_a complex
σ_3	1.489300149	0.376526076	0.067346364	$(a.u.)^{-1}h^{-1}$	Activation rate of autocatalysis
σ_4	0.027945648	0.024530964	0.02205903	h^{-1}	Rate of dissociation of FRQ:CK1 promoted by FRQ:CK1:WCC_i
σ_5	2.482894159	0.046222158	0.012197585	h^{-1}	Rate of synthesis of active WCC
K_1	2.220963035	1.362713222	0.963857481	a.u.	Threshold of critical concentration for transcription
K_2	3.073835756	1.515614232	0.562165757	a.u.	Threshold of critical concentration for transcription
K_3	0.719011473	1.145317596	3.064323196	a.u.	Threshold of critical concentration for association
K_4	0.180716273	0.941876131	0.014560894	a.u.	Threshold of critical concentration for dissociation
m	5	5	5		Hill coefficient
n	5	5	5		Hill coefficient
ℓ	5	5	5		Hill coefficient
s	5	5	5		Hill coefficient

Supplementary Table 2. Ranking of the period sensitivity of each parameter for 3 parameter sets.

	Set 1 P_0=22.0994		Set 2 P_0=22.1484		Set 3 P_0=22.13
**Parameters**	ϕ	**Parameters**	ϕ	**Parameters**	ϕ
α_1	unbounded	α_2	2.5985	α_1	unbounded
α_c	1.9259	α_1	1.6178	α_c	2.2381
α_2	1.2638	α_c	1.3554	α_2	1.2196
δ_1	0.7539	δ_1	0.5869	δ_1	0.7433
δ_5	0.3788	δ_5	0.5266	δ_5	0.2906
α_3	0.2130	δ_7	0.1645	δ_3	0.1970
δ_3	0.1444	δ_4	0.1444	δ_4	0.1057
δ_4	0.0644	δ_3	0.1142	α_w	0.0655
δ_9	0.0453	α_w	0.1135	α_3	0.0646
α_f	0.0377	α_3	0.1117	δ_7	0.0637
δ_7	0.0272	σ_1	0.0117	β_1	0.0325
α_w	0.0265	α_5	0.0110	σ_1	0.0249
β_3	0.0223	β_1	0.0110	α_f	0.0243
β_4	0.0110	β_4	0.0110	δ_8	0.0239
α_4	0.0077	α_f	0.0098	α_5	0.0218
δ_8	0.0055	β_2	0.0066	σ_3	0.0154
α_5	0	α_4	0.0044	α_4	0.0110
β_1	0	β_3	0.0044	δ_9	0.0088
β_2	0	δ_{10}	0.0033	σ_2	0.0077
δ_2	0	σ_5	0.0044	δ_{10}	0.0055
δ_6	0	σ_3	0.0011	δ_6	0.0045
δ_{10}	0	σ_4	0.0011	β_2	0.0027
σ_1	0	δ_6	0	σ_5	0.0023
σ_2	0	δ_2	0	σ_4	0.0022
σ_3	0	δ_8	0	β_3	0.0014
σ_4	0	δ_9	0	β_4	0.0005
σ_5	0	σ_2	0	δ_2	0.0005

Supplementary Table 3. List of primers used in this work.

Primer names	Sequences
FRQ-L488V-ApoI-F	GTGCTTTGTAACCTGGCCCAGCTG
FRQ-L488-N491-R	ATTGAAATTCACCCAGCCTTCCGC
FRQ-C490G-ApoI-F	CTGCTTGGTAACCTGGCCCAGCTG
FRQ-N491E-F	CTGCTTTGTGAACTGGCCCAGCTG
FRQ-L492M-PvuII-F	ATGGCCCAACTGCACATGGTTAAC
FRQ-L492M-L495V-R	GTTACAAAGCAGATTGAGATACACCCAGCC
FRQ-A493V-PvuII-F	CTGGTCCAACTGCACATGGTTAAC
FRQ-Q494N-PvuII-F	CTGGCCAACCTGCACATGGTTAAC
FRQ-V319I-F	ATTGTCCGACGCTTGGAGCAACTGTTC
FRQ-V319I-PvuII-R	CAGCTGCTTCTTCTCCTTGTCGGTCATG
FRQ-V320L-R322K-R	AACCAGCTGCTTCTTCTCCTTGTCGGTC
FRQ-V320L-F	CTGCGACGCTTGGAGCAACTGTTCACC
FRQ-V320I-F2	ATCCGACGCTTGGAGCAACTGTTCACC
FRQ-R321K-F2	GTCAAGCGCTTGGAGCAACTGTTCACC
FRQ-R322K-F2	GTCCGAAAATTGGAGCAACTGTTCACC
FRQ-L323M-PvuII-F	ATGGAGCAGCTGTTCACCGGAAAGATC
FRQ-L323M-Q325N-R	GCGTCGGACAACCAGCTTCTTCTTCTC
FRQ-E324D-PvuII-F	TTGGACCAGCTGTTCACCGGAAAGATC
FRQ-Q325N-XhoI-F	CTCGAGGGACTGTTCACCGGAAAGATC
frq T7 5UTR F	TAATACGACTCACTATAGGGGAAGCCTGCGATGCTACTCAC
frq T7 5UTR R	CATCGCATCCACTTCGCACAC
frq C-box F	GTCAAGCTCGTACCCACATC
frq C-box R	CCGAAAGTATCTTGAGCCTCC
GST-CK1-F EcoR I	GGAATTCATGACTACCATGGATCTCCGC
GST-CK1-R Sma I	TCCCCCGGGTCACATCCTGTCGCTGCCACC
